# Supplementary material for: Rapid turnover of life-cycle-related genes in the brown algae
Source: Genome Biol. 2019 Feb 14;20:35. doi: 10.1186/s13059-019-1630-6 (PMC6374913; doi:10.1186/s13059-019-1630-6)
Supplement: Supplementary file 3 — Protein alignments, corresponding Gblocks html file and CDS sequences for the lineage conserved gametophyte-biased genes. (ZIP 2109 kb) [file 13059_2019_1630_MOESM3_ESM.zip › Con_GA_alignments/Ec-26_004430.aa_ali.fasta-gb.htm]

Ec-26\_004430.aa.fasta.trans.aa\_ali.fasta


## Gblocks 0.91b Results

Processed file: **Ec-26\_004430.aa.fasta.trans.aa\_ali.fasta**  
Number of sequences: **2**  
Alignment assumed to be: **Protein**  
New number of positions: **157** (selected positions are underlined in blue)

```
                         10        20        30        40        50        60
                 =========+=========+=========+=========+=========+=========+
Ec-26_004430     --------------------------MYGGVSTEMAAEPQQQQLSG-FGEQLDGEGGAQG
MSTRG.37090.1    ADVTASDAGGEQYDPGGVMQHQPDVAIYGGASSSFGEPQQRQQPPDNFGEQPND----QG
                                            ################                 


                         70        80        90       100       110       120
                 =========+=========+=========+=========+=========+=========+
Ec-26_004430     EQGWNREEEEEEDAYEDPEEMIREFGRHPMMDRVQEALYNQLLQTYERVSEELRDKDADV
MSTRG.37090.1    EQQRG-EEEEEEDEYEDPEEMIREFGRHPMMDRVQEALYNQLLQTYERVSEELRDKDTDV
                       ######################################################


                        130       140       150       160       170       180
                 =========+=========+=========+=========+=========+=========+
Ec-26_004430     KKLRKRREFVGVELYAMQQQLAKLQVALEQTHADFNGLGEGRARAELDADAAKQRYFELK
MSTRG.37090.1    KKLRKRREFVGVELYGMQQQLAKLQVALEQTHADFNGLGEGRARAELESDAAKRRYSELK
                 ############################################################


                        190       200       210       220       230       240
                 =========+=========+=========+=========+=========+=========+
Ec-26_004430     GVGDALSKRLIKNESELNSLQDTLRQVELYNEEMKDEILVTRRATYKAEEAVSNLEKDKG
MSTRG.37090.1    GVGDALSKRLLKNEADLNSLQDTLRQV---------------------------------
                 ###########################                                 


                        250       260       270       280       290       300
                 =========+=========+=========+=========+=========+=========+
Ec-26_004430     RQDLLIDGLMDQVKRLQGQVAVAEAQIGMSQGQTGEADGMLRETAHEMSLIAFEKKQLLQ
MSTRG.37090.1    ------------------------------------------------------------
                                                                             


                        310       320       330       340       350       360
                 =========+=========+=========+=========+=========+=========+
Ec-26_004430     QWKGALVGLARRDEALTAASNALKEAQSAARDYDIEIEGLKRETLKAQEEHEGLIAVRDR
MSTRG.37090.1    ------------------------------------------------------------
                                                                             


                        370       380       390       400       410       420
                 =========+=========+=========+=========+=========+=========+
Ec-26_004430     LESGRAFVEEQLAKTKEERDSLAERYAMLQRSLAQSEEDEEKLDAMGTQLRSQVDMLSNQ
MSTRG.37090.1    ------------------------------------------------------------
                                                                             


                        430       440       450       460       470       480
                 =========+=========+=========+=========+=========+=========+
Ec-26_004430     AQALTRERQKLEAEAAKRRSQQSTTNKAVKNLVRQARAVVEQLHEIEMEEANLQNEEARV
MSTRG.37090.1    ------------------------------------------------------------
                                                                             


                        490       500       510       520       530       540
                 =========+=========+=========+=========+=========+=========+
Ec-26_004430     RVDSLNTEAHNLQLKETLRRSEEELNSKDRLIEKYQTEIRQRGDEIEKKMYSVDRLNRQY
MSTRG.37090.1    ------------------------------------------------------------
                                                                             


                        550       560       570       580       590       600
                 =========+=========+=========+=========+=========+=========+
Ec-26_004430     EKLREAVEEPENVGPLESSVKSLQKEIAALGEENARMKHEWLADQTKLVSSAMETEQLAE
MSTRG.37090.1    ------------------------------------------------------------
                                                                             


                        610       620       630       640       650       660
                 =========+=========+=========+=========+=========+=========+
Ec-26_004430     KNGELRARCSIVGQRKARLLQSMTSDLAESKRLRSAIEGMHMDMGRLNDLIGKSGRRAHE
MSTRG.37090.1    ------------------------------------------------------------
                                                                             


                        670       680       690       700       710       720
                 =========+=========+=========+=========+=========+=========+
Ec-26_004430     LDDATRALEREFVQELRDLEAESVALEGRIRSVKDLKAETLEQILEAERQVMLWEKKTQL
MSTRG.37090.1    ------------------------------------------------------------
                                                                             


                        730       740       750       760       770       780
                 =========+=========+=========+=========+=========+=========+
Ec-26_004430     ERETQAALDPGVGKSEVRTMEREIHRMRIRLEGLKREQERMIQEMERAIAKREQIAVRHK
MSTRG.37090.1    ------------------------------------------------------------
                                                                             


                        790       800       810       820       830       840
                 =========+=========+=========+=========+=========+=========+
Ec-26_004430     GKSEAAAGRSTKARRGMLASGNGAAGSGLGDLTTTALKKKVVSLQRQLNASAEETLRYTR
MSTRG.37090.1    ------------------------------------------------------------
                                                                             


                        850       860       870       880       890       900
                 =========+=========+=========+=========+=========+=========+
Ec-26_004430     AAEDRRNEVGALSSSLQEQAARADETARRGEELRSSINSSLYEKQRLAEVTAVKQRLADN
MSTRG.37090.1    ------------------------------------------------------------
                                                                             


                        910       920       930       940       950       960
                 =========+=========+=========+=========+=========+=========+
Ec-26_004430     LRGVAKGVMKPVPAHDAMKVEQALLTSEIRLGAVRDVIESMAEKFPHLRDVLGRLSRLSE
MSTRG.37090.1    ------------------------------------------------------------
                                                                             


                 
                 ====
Ec-26_004430     DATD
MSTRG.37090.1    ----
```

```
Parameters used
Minimum Number Of Sequences For A Conserved Position: 2
Minimum Number Of Sequences For A Flanking Position: 2
Maximum Number Of Contiguous Nonconserved Positions: 8
Minimum Length Of A Block: 10
Allowed Gap Positions: None
Use Similarity Matrices: Yes
```

```
Flank positions of the 2 selected block(s)
Flanks: [28  43]  [67  207]  

New number of positions in Ec-26_004430.aa.fasta.trans.aa_ali.fasta-gb:  157  (16% of the original 964 positions)
```
